# Magnetic routing of light-induced waveguides

**DOI:** 10.1038/ncomms14452

**Published:** 2017-02-15

**Authors:** Yana Izdebskaya, Vladlen Shvedov, Gaetano Assanto, Wieslaw Krolikowski

**Affiliations:** 1 Laser Physics Center, Research School of Physics and Engineering, Australian National University, Canberra, Australian Capital Territory 0200, Australia; 2 NooEL—Nonlinear Optics and OptoElectronics Lab, University of Rome ‘Roma Tre', Rome I-00146, Italy; 3 Optics Laboratory, Tampere University of Technology, Tampere FI-33101, Finland; 4 Science Program, Texas A&M University at Qatar, P.O. Box 23874, Doha, Qatar

## Abstract

Among photofunctional materials that can be employed to control the propagation of light by modifying their properties, soft dielectrics such as nematic liquid crystals (NLCs) stand out for their large all-optical response. Through reorientation, the molecular distribution of NLCs can be modified by the electric field of light, permitting functional operations and supporting self-localized light beams or spatial optical solitons. To date, the generation and routing of such solitons have been limited by the boundary conditions employed to tailor the properties of NLCs in planar cells or capillaries. Here we report on spatial solitons in bulk NLCs with no lateral anchoring, where the application of an external magnetic field effectively controls the direction of propagation and the angular steering of the self-trapped wavepackets. Our results entail a completely new approach to the routing of self-localized beams and light-induced waveguides in three dimensions, without the usual limitations imposed by transverse boundary conditions.

## Abstract (web summary)

Nematic liquid crystals are frequently used as a reconfigurable material to control light propagation and as a nonlinear medium supporting solitons. Here, the authors demonstrate steering of such solitons in bulk nematic liquid crystals without lateral anchoring by external magnetic fields.

The main interest in practical applications of optical spatial solitons is caused by their ability to serve as light-induced channels for optical information and, therefore, as basic elements for all-optical signal processing^1^,^2^,^3^. The latter requires soliton waveguides to be reconfigurable in space by modifying the soliton trajectories through external stimuli. Developing efficient strategies to achieve such control is one of the challenges in nonlinear optics^4^. To date, the control of soliton trajectories has been achieved only over small steering angles in bulk solid-state media, such as photorefractive crystals^5^,^6^, or in specific planar geometries in soft matter, such as liquid crystals^7^,^8^,^9^,^10^,^11^,^12^,^13^. Photorefractive crystals allow for varying soliton path, but are rather expensive, require usage of high voltages and have relatively long relaxation times^5^,^6^,^14^. Conversely, liquid crystals are affordable and efficient because of their unique sensitivity to light–matter interactions^15^,^16^,^17^. The key to such sensitivity is the organization of nematic liquid crystal (NLC) anisotropic molecules, providing a reorientational response to the electric field of incident light^16^. The ensuing nonlinearity and the formation of spatial solitons normally require appropriate molecular alignment at the boundaries, with a well-defined orientation of the optical axis^8^,^15^. Hence, the steering of spatial solitons in NLCs, so-called nematicons^7^, has been limited by cell geometries, such as hollow channels^18^ or planar cells with an NLC layer, typically 0.1 mm or less in thickness^8^,^19^. The most notable experimental results in nematicon steering have been reported in planar configurations^9^,^20^,^21^,^22^, whereby molecular orientation is defined by anchoring on the cell glass/NLC interfaces^19^. In planar cells the NLC alignment can be modified by applying a low-frequency voltage across the layer by means of thin film or solid electrodes^8^,^9^,^23^, by changing the beam power^13^,^24^, by using additional beams^25^,^26^ or by photoalignment layers^10^,^27^,^28^. However, fully three-dimensional (3D) soliton dynamics is hampered by the dimensional restrictions of these planar structures^29^, as beam trajectories can only vary within the NLC region about the mid-plane of the cell, with angular steering not exceeding fractions of degree orthogonally to the propagation plane parallel to the interfaces^8^,^9^.

In this paper we demonstrate three-dimensional steering of self-trapped beam paths by employing bulk NLC samples without lateral boundary conditions and a magnetic field to modify their molecular orientation. In achieving this goal, we manage to exploit magnetic molecular reorientation^16^ while preserving the nonlinear optical response of the material, introducing magneto-optic control of spatial solitons, realizing 3D soliton routing without limitations of voltage-controlled planar schemes, and hence paving the way to a whole new family of signal routers and processors.

## Results

### Operation with external magnetic field

**Figure 1:**
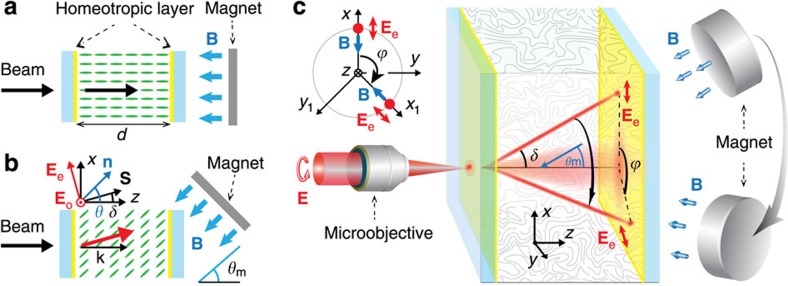
Basic configuration of the magnetic routing of self-induced waveguides in nematic liquid crystal. A circularly polarized laser beam propagates along the *z* axis, normally to the glass slide-NLC interface and focuses into the NLC bulk. The homeotropic layers (shaded in yellow) on the inner surfaces of input and output glass slides ensure an initial alignment of the NLC molecular director predominantly along *z*. Such homeotropic orientation can be (**a**) strengthened or (**b**) altered by applying an external magnetic field **B** at a polar angle *θ*_m_ with respect to *z,* reorienting the molecular director **n** at a new polar angle *θ*. For *θ*_m_≠0 an extraordinary light beam with electric vector **E**_e_ can self-trap into a soliton while propagating with walk-off *δ* between its Poynting vector **S** and its wavevector **k**||**z**. (**c**) 3D angular steering of an extraordinary polarized soliton by varying the orientation of the magnetic field. A rotation of the magnetic field at the azimuthal angle *ϕ* modifies the soliton direction by the same angle.

The experimental geometry is sketched in Fig. 1. Unlike in earlier reports^7^,^9^, in the adopted configuration (see Methods) the sample was not subjected to boundary conditions on the sides (orthogonally to *x* and *y*), but exclusively at input and output facets orthogonal to the direction of propagation *z* of the incident light beam.

As we discuss below, the orientation of the NLC molecular director **n** (that is, the optical axis of the corresponding uniaxial medium) with respect to *z* can be adjusted in the bulk by applying an external magnetic field^16^. Throughout this work we assume that the magnetic field is sufficiently strong to completely reorient the molecular axes along the vector **B** in the absence of optical excitations. We consider a uniform field strength and, as a result, a homogeneous distribution of **n** in the sample. Although some line disclinations of the director may occur when rotating the field **B** with respect to *z* in the proximity of input and output interfaces, these are not expected to affect the bulk optical properties of NLCs. In our experiments we used cylindrical permanent magnets (20 mm diameter) placed at a distance of 10 mm from the entrance point of the beam. Considering that the magnet size is relatively big in comparison with the separation between the magnet and the beam path, the magnetic field could be assumed uniform at the propagation distance of the beam in the bulk NLC (limited to *d*=1 mm in our sample). The magnetic field strength measured at this distance was *B*=0.2 T.

### Soliton formation

Let us consider a monochromatic Gaussian beam of wavelength *λ* and a given direction of propagation with wavevector **k** along the *z* axis. At optical frequencies, NLCs behave as a typical birefringent uniaxial medium with positive dielectric anisotropy Δ*ɛ*=n^2^_∥_−n^2^_⊥_, and refractive indices n_⊥_ and n_∥_ (n_⊥_<n_∥_) for electric fields of light perpendicular or parallel to the molecular director **n**, respectively. With proper anchoring at input and output interfaces to ensure a homeotropic orientation, the initial orientation of the NLC director **n**_0_(*x,y,z*), in the absence of a magnetic field and light, is set at the background value *θ*_b_(*x,y,z*)=0 with respect to the *z* axis. Denoting by *θ*_m_ the polar angle between *z* and the applied magnetic field vector and assuming complete magnetic reorientation, the resulting optical axis **n** takes a new background orientation at *θ*_m_. Hence, the light beam splits into two independent linearly polarized components, namely an ordinary (o) wave beam with electric field *E*_o_ orthogonal to the plane **kn** and an extraordinary (e) wave with the electric field ***E***_e_ coplanar with **k** and **n**.

While NLCs are insensitive to the ordinary o-polarized beam below the so-called Freedericks transition^16^, the electric field **E** of the extraordinary e-polarized beam exerts an optical torque on the highly polarizable NLC molecules, which tends to align the director along the electric field vector. The torque tilts the director by a small (in the weak nonlinear regime) angle *ψ* in the region around the beam axis (at peak intensity), and the local director orientation becomes *θ*=*θ*_m_+*ψ*, with *θ*_m_ the uniform distribution of the director in the absence of optical stimuli. The extraordinary index 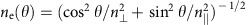 increases in the region surrounding and containing the beam, inducing a graded-index waveguide and allowing for self-confinement^7^,^18^,^19^. As the trapped beam propagates with wavevector 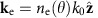, the Poynting vector tilts at the walk-off angle 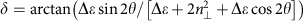 with respect to **k**_e_ due to birefringence.

### Soliton routing

The propagation direction of the e-polarized soliton-forming beam in our geometry is determined by the spatial orientation of the stationary magnetic field **B**. An arbitrary fixed direction of the magnetic vector defines both the director **n** and the walk-off orientation, with respect to chosen coordinates. While the *z* axis is always orthogonal to the entrance interface of the NLC and 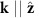, the axes *x* and *y* with corresponding unit vectors 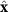 and 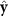 can be chosen arbitrarily in the plane *z*=const. Let us assume that the **B** vector has fixed polar orientation with an angle *θ*_m_ and is initially coplanar with the *xz* plane, so 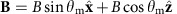, as shown in Fig. 1c. After rotating by an azimuthal angle *ϕ* around the *z* axis the magnetic field becomes coplanar with the new plane *x*_1_*z*: 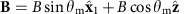 in a new coordinate basis 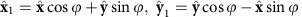. As the molecular director **n** together with the electric field of extraordinary beam **E**_*e*_ follow the plane *zB*, the trajectory of spatial soliton lies in the planes *yz* and *y*_1_*z* before and after the rotation, respectively. In our experimental conditions the effect of optical torque on the polar director orientation *θ* is significantly small (*ψ*<<*θ*_m_) in comparison with the magneto-induced torque as we are in the weak nonlinear regime^30^. Hence, the resulting walk-off angle *δ* is practically determined by *θ*_m_:


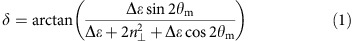


In this equation models well our experimental observations of magnetic-field control of spatial solitons in bulk liquid crystals with no lateral boundary conditions. The model neglects the interplay between all-optical and magnetic reorientations, while describing the walk-off of a bell-shaped beam on axis, that is, in the framework of the highly nonlocal approximation^7^,^30^,^31^,^32^. In other words, we take a linearized superposition of all-optical and magnetic-driven orientations, analogous to the nonlinear optical response in the presence of an external voltage bias^7^,^19^.

## Discussion

We used circularly polarized beams (*λ*=800 nm) yielding o- and e-components of equal powers in the NLC mixture 6CHBT (n_⊥_=1.5144 and n_∥_=1.6714 at 20 °C), independently of *θ*.

While the electric field **E**_e_ of the e-polarized beam tends to align NLC molecules along its own direction, the magnetic field determines the background alignment; the stronger the **B**, the higher the beam intensity/power required to produce an index change 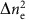 for self-trapping. Hence, equation (1) is valid as long as the external magnetic field effects are comparable with those due to the electric field of light. In this perturbative regime, we observed diffractionless propagation of ∼6 μm diameter solitons over millimetre distances, at powers from a few to tens of mW depending on the magnetic field direction.

**Figure 2:**
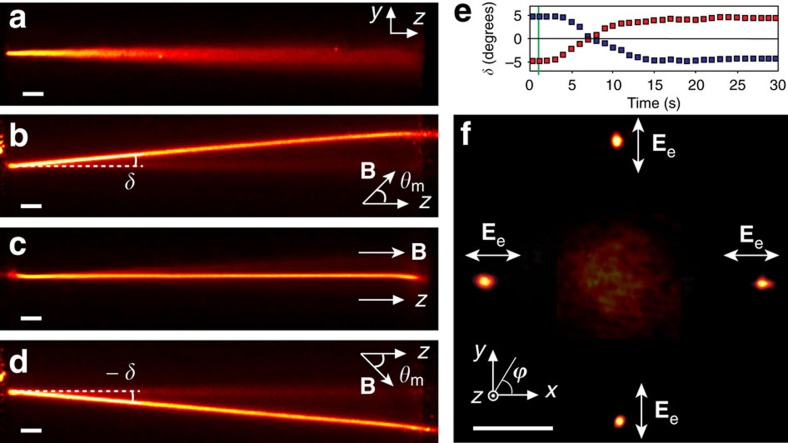
Soliton steering in NLC by means of an external magnetic field. (**a**) Acquired images of an ordinary wave beam undergoing diffraction while propagating along the *z* axis, (**b**–**d**) projections of trajectories of extraordinary-wave self-confined solitons (visible because of weak out-of-plane light scattering) on the *zy* plane, from a circularly polarized beam of power *P*=7 mW when **B** is oriented at a fixed polar angle *θ*_m_=45° with respect to *z* and various azimuthal angles *ϕ*: 90° (**b**), 0°, 180° (**c**) and 270° (**d**) with respect to *x*, respectively. (**e**) Time evolution of the beam walk-off in the plane *yz* when the orientation of the magnetic field (*B*=0.2T) was abruptly (<1 s) switched from the angular position *θ*_m_=45°, *ϕ*=90° to *θ*_m_=45°, *ϕ*=270° (red squares) and from *θ*_m_=45°, *ϕ*=270° to *θ*_m_=45°, *ϕ*=90° (blue squares), respectively. The vertical green line indicates when **B** was switched. (**f**) Intensity profiles of the output beam for various values of *ϕ*. The broad spot in the centre corresponds to the diffracting ordinary-wave beam; the four narrow spots are the outputs of extraordinary-wave solitons, angularly displaced by the orientation of the magnetic field. Counterclockwise from right: *ϕ*=0°, 90°, 180°and 270°, respectively. The white scale bars in **a**–**d**,**f**, 50 μm.

Figure 2 shows the experimental results on soliton generation and routing. Beam trajectories (Fig. 2a–d) and output intensity profiles (Fig. 2f) are reported for a circularly polarized fundamental Gaussian input. When the magnetic field **B** is applied parallel to the input beam wavevector (*θ*_m_=0) or is absent, the low-power beam (*P*=7 mW) rapidly diffracts (Fig. 2a). When the magnetic field is tilted with respect to the *z* axis (*θ*_m_≠0), the optical wavepacket splits into ordinary and extraordinary-wave components. The former still propagates along the original direction (*z* axis) experiencing diffraction, while the latter propagates at the walk-off angle *δ* and forms a spatial soliton via reorientation-induced self-focusing. The evolution of the self-localized beam in our geometry depends on the direction of the magnetic field. As the magnetic field rotates by an angle *ϕ* around the *z* axis, the beam trajectory follows this rotation and remains coplanar with them in the plane *zB* (see Fig. 1c). Figure 2b–d,f shows soliton trajectories for *θ*_m_=45° with *ϕ*=90°, 0°, 180°, 270° in the planes *yz* (side view) and *xy* (output), respectively. Here the circularly polarized input beam had a total power of 7 mW, the e-wave soliton ∼3.5 mW with its polarization in the plane *zB*, as only extraordinary waves could contribute to all-optical reorientation. Hence, we verified that the orientation of the magnetic field determined the principal plane for the e-wave component of the beam undergoing reorientational self-confinement.

**Figure 3:**
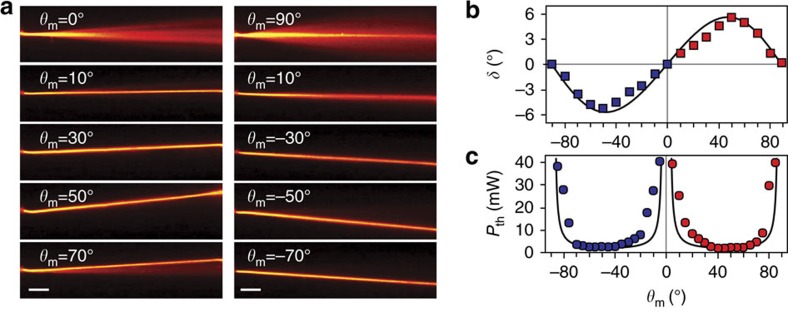
Effect of magnetic field orientation on soliton trajectory. (**a**) Photographs of soliton trajectories in the fixed *yz* plane with walk-off determined by the magnetic field orientation *θ*_m_. The white scale bar, 100 μm in each photograph. (**b**) Measured walk-off versus *θ*_m_. The solid line is calculated from equation (1). (**c**) Threshold power for soliton formation versus *θ*_m_. The solid line shows the qualitative trend *P*_th_=*P*_0_/sin 2*θ*_m_, with *P*_0_=1.9 mW. The blue and red markers in **b**,**c** correspond to the negative and positive walk-off angles in the *yz* plane, respectively.

Soliton formation strongly depends on both the angular direction *θ*_m_ of the magnetic field and the power of the incident light beam. Figure 3 illustrates the role of *θ*_m_ on the wavepacket walk-off and the soliton threshold power. We measured the walk-off by varying *θ*_m_ from −90° to +90° in the plane *yz* and obtained results (Fig. 3b) in perfect agreement with equation (1).

In order to measure the soliton threshold power *P*_th_, the input beam was set to be an extraordinary-wave with electric field coplanar to the *zB* plane. The results are plotted in Fig. 3(c). The lowest experimental threshold was 1.9±0.2 mW for a magnetic field oriented around *θ*_m_=45° with respect to *z*. The experimental data follow closely the theoretical dependence of the soliton threshold on the orientation *θ*_m_. The self-trapping threshold power, in fact, is approximately proportional to the inverse of the nonlinear index change^1^. The latter, in turn, depends on sin2*θ*_m_ (ref. 7), leading to *P*_th_∝1/sin 2*θ*_m_ as indicated by the solid line in Fig. 3(c).

We also observed unstable self-confinement even for *θ*_m_ approaching 0° and 90°, but at much higher powers (>40 mW). In both situations the beam experiences fluctuation of its trajectory near the *z* direction as well as temporal transitions between self-focusing and diffraction regimes due to the Freedericks transition threshold^16^,^33^,^34^ or competition between reorientational and thermal responses through residual absorption^35^,^36^. While the self-confined beam for *θ*_m_ approaching 90° was observed in a linear state of polarization along the magnetic field, the self-confinement for *θ*_m_=0° had a random polarization accompanying symmetry-breaking instabilities^34^. A detailed analysis of these unstable cases will be carried out in future work.

**Figure 4:**
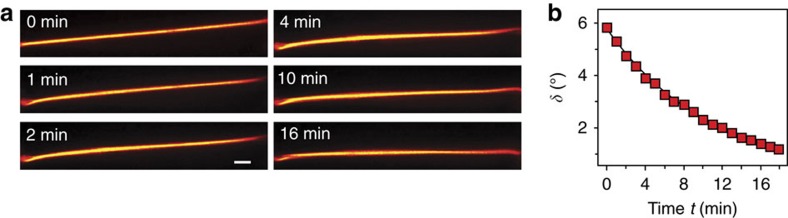
Time relaxation of the soliton path. The magnetic field had been oriented at the angle *θ*_m_=50° in the *yz* plane before it was switched off at the moment *t*=0 min. (**a**) Photographs of time sequence of soliton trajectories in the *yz* plane. The white scale bar, 100 μm. (**b**) Measured temporal evolution of walk-off angle (squares). The solid line is the exponential fit *δ*=*δ*_0_ exp(−*t*/*τ*), with *τ*=11 min.

We also evaluated the time required by self-confined beams to relax to their original trajectories once the magnetic field was switched off, as illustrated in Fig. 4 for a 7 mW incident beam power. The plot in Fig. 4b shows an exponential decay *δ*=*δ*_0_ exp(−*t*/*τ*) of the walk-off angle from its maximum value *δ*_0_=5.8° at *θ*_m_=50°.

The measured relaxation time *τ*=11 min needs be compared with the theoretical estimation of director relaxation time^37^ *τ*_*r*_=*γd*^2^/*Kπ*^2^ of a homeotropically aligned NLC. For cell thickness *d*=1 mm on the basis of the known rotation viscosity coefficient *γ*≈16 mPa s and elastic constants *K*∼10 pN of the liquid crystal 6CHBT^38^,^39^, *τ*_*r*_≈2.7 min. The discrepancy can be attributed to the fact that the theory is based on long-range mean field potential^40^ in thin (<100 μm) films of NLC. In our thick NLC sample (1 mm) geometry, the short-range intermolecular correlations apparently play a significant role in the molecular reorientations and the field turn-off relaxation becomes a slower process. The relaxation time is mostly determined by the director behaviour in the cell's middle layers, since the restoration torque, which returns molecular orientations to their equilibrium positions, mainly comes from the homeotropic anchoring boundaries once the magnetic field is switched off.

Nevertheless, the beam path dynamics mediated by the magnetic field is rather fast (Fig. 2e). As the applied magnetic field rapidly changes its orientation, the molecular director in the LC middle layers experiences magnetic torque, which results in an accelerated relaxation time. When the magnetic field direction was changed (in≈1 s) from *θ*_m_=−45° to *θ*_m_=45°, the soliton trajectory adjusted within 18 s, a time delay much shorter than obtained by photo-orientation^27^ and comparable with voltage-driven switching in planar geometries^7^,^8^,^9^.

In summary, employing a bulk NLC geometry with neither lateral anchoring nor external voltage, we demonstrated that optical spatial solitons can be generated and routed by applying a moderate magnetic field. The magnetic field in such a bulk configuration provides fully controllable 3D molecular orientation, entailing extra flexibility when redirecting self-confined light beams and re-addressing all-optical waveguides in quasi-static layouts. The substantial freedom from lateral bounds and corresponding anchoring conditions unveils rich scenarios for soliton-based optical circuits and reconfigurable photonic interconnects with the aid, for example, of electrically driven coils.

## Methods

### Sample preparation

The planar cell used in our experiments consists of two parallel optical grade glass slides placed normal to the *z* axis, measuring 10 × 10 mm across *x* and *y*: such dimensions along both the transverse coordinates prevented boundary effects. The slide surfaces (at the glass–NLC interfaces) were chemically treated to induce homeotropic molecular alignment, thus ensuring a homogeneous distribution with **n** parallel to the *z* axis in the absence of external stimuli. The slide treatment also avoided undesired beam depolarization.

We employed the NLC mixture 6CHBT. Its thickness *d*=1 mm along *z* was defined by rectangular spacers, so that we could observe the beam output position and its profile despite scattering. The two leak-proof spacers were attached using ultraviolet curable glue. To seal the cell, two additional 100-μm-thick glass slides were attached to the other two edges and served as side-windows. A small aperture was left at the top edge to permit cell filling by capillarity.

### Experimental set-up

We injected either circularly or linearly extraordinary-wave polarized Gaussian beams (fundamental mode) of wavelength *λ*=800 nm and waist *w*≈3 μm using a 0.45 numerical aperture dry microscope objective (20 ×). The orthogonality between the input wavevector and the NLC–glass input interface was ensured within ∼0.1° by measuring back-reflection. The evolution of the beam trajectory in 3D was imaged through the observation side-windows with two optical microscopes, collecting the light scattered by the NLC out of the planes *xz* and *yz*, respectively. The output profile and position of the beam were imaged by a third optical microscope collecting light at the exit facet (plane *xy*). A cylindrical permanent neodymium magnet (10 mm thick and 20 mm in diameter), mounted on a six-dimensional mechanical stage, was used to control the molecular orientation. The magnet axis was always pointed at the beam entrance in order to avoid potential artefacts due to field inhomogeneities and edge effects. The magnetic field strength was constant (*B*=0.2 T) in the volume of the NLC. The beam trajectories in the NLC bulk were acquired for several launch positions in order to average out contributions from noise and imperfections at the entrance facet.

### Data availability

The authors declare that the data supporting the findings of this study are available within the paper. All relevant additional data are available from the authors.

## Additional information

**How to cite this article:** Izdebskaya, Y. *et al*. Magnetic routing of light-induced waveguides. *Nat. Commun.* **8,** 14452 doi: 10.1038/ncomms14452 (2017).

**Publisher's note:** Springer Nature remains neutral with regard to jurisdictional claims in published maps and institutional affiliations.

## References

[b1] Kivshar Y. U. S. & Agrawal G. P. in Optical Solitons: from Fibers to Photonic Crystals 1st edn. Academic Press (2003).

[b2] Snyder A. W. & Ladouceur F. Light guiding light: letting light be the master of its own destiny. Opt. Photon. News 10, 35–39 (1999).

[b3] Hasegawa A. & Kodama Y. in Solitons in Optical Communications 1st edn. Clarendon Press (1995).

[b4] Kivshar Y. U. S. & Stegeman G. I. Spatial optical solitons: guiding light for future technologies. Opt. Photon. News 13, 59–63 (2002).

[b5] Crosignani B. & Salamo G. Photorefractive solitons. Opt. Photon. News 13, 38–41 (2002).

[b6] Duree G. . Observation of self-trapping of an optical beam due to the photorefractive effect. Phys. Rev. Lett. 71, 533536 (1993). 10.1103/PhysRevLett.71.533 10055300

[b7] Peccianti M. & Assanto G. Nematicons. Phys. Rep. 516, 147–208 (2012).

[b8] Peccianti M., Conti C., Assanto G., De Luca A. A. & Umeton C. Routing of anisotropic spatial solitons and modulational instability in liquid crystals. Nature 432, 733–737 (2004). 15592407 10.1038/nature03101

[b9] Peccianti M., Dyadyusha A., Kaczmarek M. & Assanto G. Tunable refraction and reflection of self-confined light beams. Nat. Phys. 2, 737–742 (2006).

[b10] Piccardi A., Assanto G., Lucchetti L. & Simoni F. All-optical steering of soliton waveguides in dye-doped liquid crystals. Appl. Phys. Lett. 93, 171104 (2008).

[b11] Alberucci A., Piccardi A., Bortolozzo U., Residori S. & Assanto G. Nematicon all-optical control in liquid crystal light valves. Opt. Lett. 35, 390–392 (2010). 20125731 10.1364/OL.35.000390

[b12] Piccardi A. . Frequency-controlled acceleration of spatial solitons in nematic liquid crystals. Appl. Phys. Lett. 101, 081112 (2012).

[b13] Piccardi A., Alberucci A., Kravets N., Buchnev O. & Assanto G. Power-controlled transition from standard to negative refraction in reorientational soft matter. Nat. Commun. 5, 5533 (2014). 25420491 10.1038/ncomms6533

[b14] Stegeman G. I. & Segev M. Optical spatial solitons and their interactions: universality and diversity. Science 286, 1518–1523 (1999). 10567250 10.1126/science.286.5444.1518

[b15] Khoo I. C. Nonlinear optics of liquid crystalline materials. Phys. Rep. 471, 221–267 (2009).

[b16] De Gennes P. G. & Prost J. in The Physics of Liquid Crystals 2nd edn. Clarendon Press (1995).

[b17] Beeckman J., Neyts K. & Vanbrabant P. Liquid-crystal photonic applications. Opt. Eng. 50, 081202–081217 (2011).

[b18] Warenghem M., Henninot J. F. & Abbate G. Nonlinearly induced self-waveguiding structure in dye doped nematic liquid crystals confined in capillaries. Opt. Express 2, 483–490 (1998). 19381219 10.1364/oe.2.000483

[b19] Peccianti M. . Electrically assisted self-confinement and waveguiding in planar nematic liquid crystal cells. Appl. Phys. Lett. 77, 7–9 (2000).

[b20] Izdebskaya Y. V., Shvedov V. G., Desyatnikov A. S., Krolikowski W. & Kivshar Y. U. S. Soliton bending and routing induced by interaction with curved surfaces in nematic liquid crystals. Opt. Lett. 35, 1692–1694 (2010). 20479852 10.1364/OL.35.001692

[b21] Barboza R., Alberucci A. & Assanto G. Large electro-optic beam steering with nematicons. Opt. Lett. 36, 2725–2727 (2011). 21765522 10.1364/OL.36.002725

[b22] Piccardi A. . In-plane steering of nematicon waveguides across an electrically adjusted interface. Appl. Phys. Lett. 100, 251107 (2012).

[b23] Izdebskaya Y. V. Routing of spatial solitons by interaction with rod microelectrodes. Opt. Lett. 39, 1681–1684 (2014). 24690868 10.1364/OL.39.001681

[b24] Piccardi A., Alberucci A. & Assanto G. Power-dependent nematicon steering via walk-off. J. Opt. Soc. Am. B 27, 2398–2404 (2010).

[b25] Fratalocchi A., Piccardi A., Peccianti M. & Assanto G. Nonlinearly controlled angular momentum of soliton clusters. Opt. Lett. 32, 1447–1449 (2007). 17546150 10.1364/ol.32.001447

[b26] Izdebskaya Y. V., Rebling J., Desyatnikov A. S., Assanto G. & Kivshar Y. U. S. All-optical switching a signal by a pair of interacting nematicons. Opt. Express 20, 24701–24707 (2012). 23187233 10.1364/OE.20.024701

[b27] Ouskova E. . Photo-orientation of liquid crystals due to light-induced desorption and adsorption of dye molecules on an aligning surface. Phys. Rev. E 64, 05709 (2001). 10.1103/PhysRevE.64.051709 11735947

[b28] Piccardi A., Alberucci A., Bortolozzo U., Residori S. & Assanto G. Soliton gating and switching in liquid crystal light valve. Appl. Phys. Lett. 96, 071104 (2010).

[b29] Alberucci A. & Assanto G. Propagation of optical spatial solitons in finite size media: interplay between non locality and boundary conditions. J. Opt. Soc. Am. B 24, 2314–2320 (2007).

[b30] Alberucci A., Jisha C. P., Smyth N. F. & Assanto G. Spatial optical solitons in highly nonlocal media. Phys. Rev. A 91, 013841 (2015).

[b31] Assanto G., Minzoni A. A. & Smyth N. F. Light self-localization in nematic liquid crystals: modelling solitons in reorientational media. J. Nonlinear Optic. Phys. Mat. 18, 657–691 (2009).

[b32] Alberucci A., Assanto G., MacNeil J. M. L. & Smyth N. F. Nematic liquid crystals: an excellent playground for nonlocal nonlinear light localization in soft matter. J. Nonlinear Optic. Phys. Mat. 23, 1450046 (2014).

[b33] Kwasny M. . Self-guided beams in low-birefringence nematic liquid crystals. Phys. Rev. A 86, 013824 (2012).

[b34] Alberucci A., Piccardi A., Kravets N., Buchnev O. & Assanto G. Soliton enhancement of spontaneous symmetry breaking. Optica 2, 783–789 (2015).

[b35] Laudyn U. A. . Nonlinear competition in nematicon propagation. Opt. Lett. 40, 5235–5238 (2015). 26565843 10.1364/OL.40.005235

[b36] Karpierz M. A., Sierakowski M., Swillo M. & Wolinski T. R. Self-focusing in liquid crystalline waveguides. Mol. Cryst. Liq. Cryst. 320, 157–163 (1998).

[b37] Yang D. K. & Wu S. T. in Fundamentals of Liquid Crystal Devices 2nd edn. Wiley (2014).

[b38] Jadzyn J., Hellemans L., Czechowski G., Legrand C. & Douali R. Dielectric and viscous properties of 6CHBT in the isotropic and nematic phases. Liq. Cryst. 27, 613–619 (2000).

[b39] Klus B., Laudyn U. A., Karpierz M. A. & Sahraoui B. All-optical measurement of elastic constants in nematic liquid crystals. Opt. Express 22, 30257–30266 (2014). 25606956 10.1364/OE.22.030257

[b40] Shiyanovskii S. V. & Lavrentovich O. D. Dielectric relaxation and memory effects in nematic liquid crystals. Liq. Cryst. 37, 737–745 (2010).

